# Urban crime prediction based on spatio-temporal Bayesian model

**DOI:** 10.1371/journal.pone.0206215

**Published:** 2018-10-31

**Authors:** Tao Hu, Xinyan Zhu, Lian Duan, Wei Guo

**Affiliations:** 1 State Key Laboratory of Information Engineering in Surveying, Mapping and Remote Sensing, Wuhan University, Wuhan, China; 2 Collaborative Innovation Center of Geospatial Technology, Wuhan University, Wuhan, China; 3 Department of Geography, Kent State University, Kent, Ohio, United States of America; 4 Key Laboratory of Environment Change and Resources Use in Beibu Gulf, Guangxi Teachers Education University, Guangxi, China; 5 Geography Science and Planning School, Guangxi Teachers Education University, Guangxi, China; University College London, UNITED KINGDOM

## Abstract

Spatio-temporal Bayesian modeling, a method based on regional statistics, is widely used in epidemiological studies. Using Bayesian theory, this study builds a spatio-temporal Bayesian model specific to urban crime to analyze its spatio-temporal patterns and determine any developing trends. The associated covariates and their changes are also analyzed. The model is then used to analyze data regarding burglaries that occurred in Wuhan City in China from January to August 2013. Of the diverse socio-economic variables associated with crime rate, including population, the number of local internet bars, hotels, shopping centers, unemployment rate, and residential zones, this study finds that the burglary crime rate is significantly correlated with the average resident population per community and number of local internet bars. This finding provides a scientific reference for urban safety protection.

## Introduction

Crime is patterned, decisions to commit crimes are patterned, and the process of committing crimes are also patterned [1]. For example, repeat and near-repeat phenomenon has been explored for burglaries, whereby risks cluster in space and time [2–4]. With this phenomenon, it is possible for the police to know when resources are best allocated to an individual location and for how long, and when resources should be allocated to a local area [2]. Thus, analyzing crime patterns and predicting crime trends is crucial to reducing the rate of revictimization. There are many studies considering local variations in crime changes in space and time scale while predicting future crimes. However, while measuring crime trends at the small-area scale, traditional crime reporting methods do not address the small number problem, resulting in a tendency for small variations in crime count to have large impacts on the crime rate. The aim of this paper is to introduce a spatio-temporal Bayesian model, examples of which have been used to model disease propagation, to investigate the development and spatio-temporal characteristics of local crime at the small-area level, providing a scientific reference for formulating a burglary prevention and control strategy.

In this case study, burglaries that occurred in Jianghan District, Wuhan City from January to August 2013 are selected. A spatio-temporal Bayesian model is used to analyze the spatio-temporal distribution patterns based on historical data, to explore the relationships between crimes and socio-economic variables (e.g., the presence of internet bars, hotels, unemployment rate, and residential zones), and to illustrate the trends in burglaries. The insights obtained from this model provide important references for urban crime prediction and management.

The remainder of the paper is structured as follows. Section 2 reviews existing research on spatial-temporal Bayesian models. Section 3 describes the study area and data used. Section 4 establishes the crime oriented spatial-temporal Bayesian model based on binomial distribution and Poisson distribution. Section 5 presents crime exploratory analysis and prediction results. Finally, Section 6 reflects on the results and suggests directions for future work.

## Related work

The study of the geography of crime came of age in the 1980s, and the quickening of pace in terms of research, as well as the willingness to move into new kinds of topical areas, reflect this [5]. Some studies explore crime hotspots to predict spatial patterns [6–11]; some of them explore the relationships between criminal activity and socio-economic variables, such as education, ethnicity, income level, and unemployment [12–15]. However, those works do not pay sufficient attention to the spatial-temporal element [16].

Cluster and hotspot detection methods are popular in the spatial-temporal analysis, such as the Knox test, Kulldorff space-time scan, and Jacquez test. Johnson et al. provided new insights into the spatial and temporal distribution of repeat victimization in 1997. Based on the examination result, it can be observed that the rate of repeat victimization was higher than that expected on the basis of statistical likelihood and that the time course of repeat victimization conformed to an exponential model [3]. Later, Johnson and Bowers analyzed time and location relative to a burgled home to identify methods to prudently allocate crime reduction resources in the wake of an offence [4]. Following this research, Johnson compares the ubiquity of the near-repeat phenomenon by analyzing space-time patterns of burglary in 10 areas, located in five different countries [2]. Grubesic and Mack explore the utility of statistical measures for identifying and comparing the spatio-temporal footprints of robbery, burglary, and assault, and suggest that these three types of crimes have dramatically different spatio-temporal signatures [17]. However, most of these methods detect clusters or hotspots and identify risk factors through traditional spatial statistical models and these frequentist cluster techniques do not account for the small number problem.

Many other scholars have studied the spatio-temporal regularities of crime as well [18–22] and the popular methods include group-based trajectory analysis, conditional spatial Markov chains, and agent-based modeling. Group-based trajectory analysis divides the crime data into different spatial groups and then studies the trajectories of the group statistics, thus predicting crime trends. Conditional spatial Markov chains are used to study the shift in crime space density over different time periods [22]. Based on routine activity theory, agent-based modeling provides an information feedback mechanism and possesses dynamic spatio-temporal characteristics. This approach can be used for spatio-temporal simulation and the prediction of important crime issues [23]. For instance, to predict the number of crimes in Pittsburg, USA, over the short term, Gorr et al. partitioned the urban space into a grid and applied a time sequence forecast method to each cell of the grid separately [24]. Using a group-based trajectory analysis method, Groff et al. studied block-level spatial trajectories of crime in Seattle, USA, and tried to find spatial changes in crime by analyzing the spatial distribution patterns of blocks with similar trajectories [25]. Despite its high practical value, group-based trajectory analysis does not consider spatial correlation and does not handle significant variations within a small region well.

In spatial statistics, spatial regression methods are used to quantify the relative influence of factors on health, crime, etc. Spatial lag and spatial error models [26] are popularly adopted in spatial regression analysis. However, these models assume that dependent variables are continuous and normally distributed, and require that parameters should be non-random variables, failing to process or analyze available information systematically. In contrast, the Bayesian spatial regression model treats data as fixed and unknown quantities or parameters as random variables expressed in terms of probabilities; thus, it can leverage information on the adjacent regions to estimate the dependent variables, overcoming the data sparseness and small-area problem, and making the estimation results more stable. Law et al. first used the Bayesian modeling approach to analyze the trend over time of lost property cases in different local regions of York City, Canada [27]. Taking spatio correlation and variation into account, the Bayesian modeling approach was able to predict the general trend for lost property and its variations in different local regions [28].

Nevertheless, these studies include only spatial parameters and do not recognize temporal variability. For Bayesian spatial models, it is convenient to process observations from more than one location and time period, integrating spatial, temporal, and spatial-temporal interaction information. Therefore, Bayesian spatio-temporal modeling approaches—specifically approaches that employ spatial and temporal random effects to analyze local patterns over time—are popularly used in spatial epidemiological studies. Spatio-temporal Bayesian modeling is used to study the mapping of disease distribution over a small region, geographic clusters of disease, and the correlation of diseases [29]. Considering that crime risks have a certain similarity to the infection of epidemic diseases, spatio-temporal Bayesian modeling is of great practical and potential value in the spatio-temporal analysis of crimes.

## Study area and data

### 3.1. Study area

**Fig 1:**
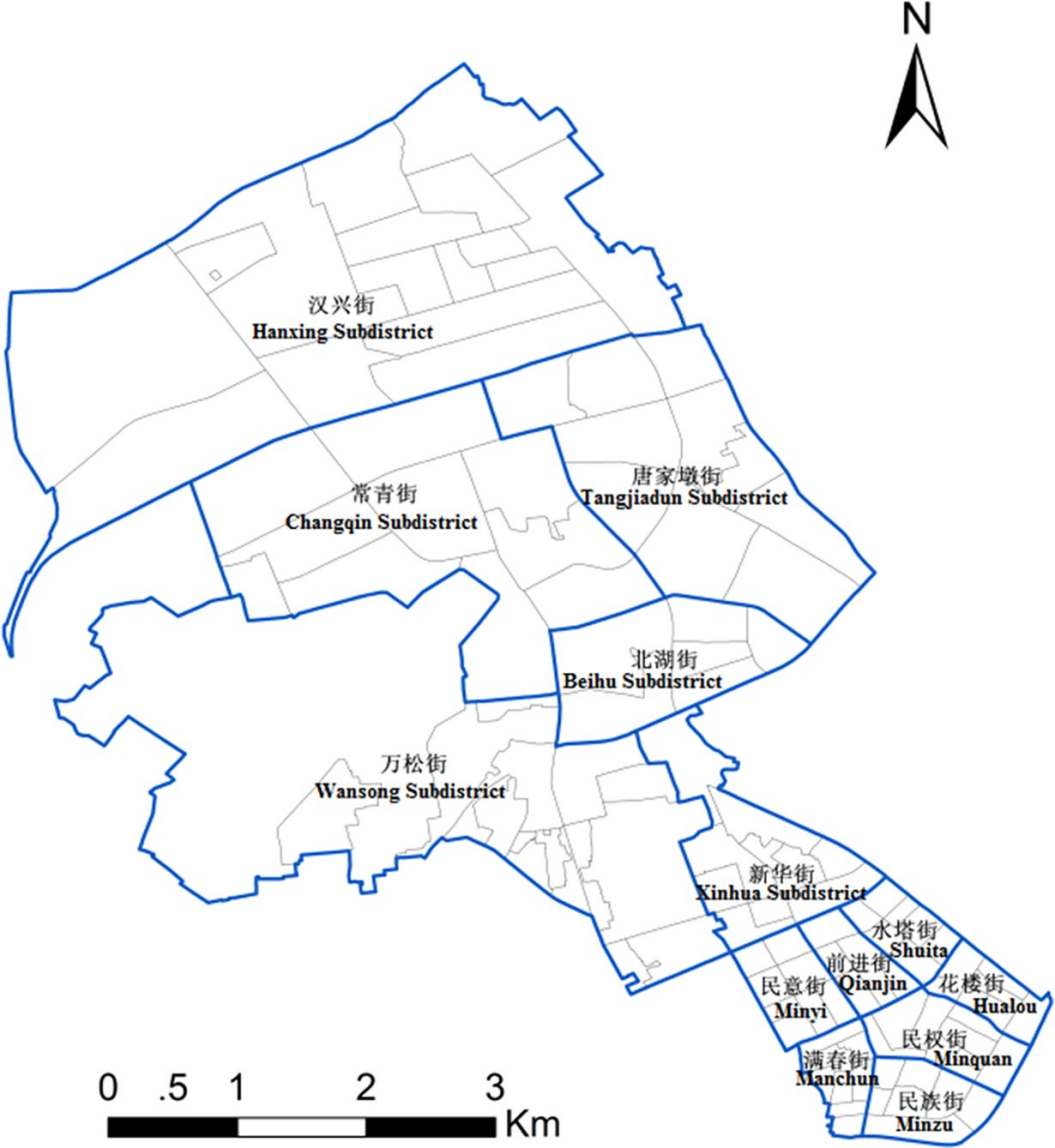
Sub-districts and communities of Jianghan District.

The city in this research is Wuhan, the largest sub-provincial city in central China. As of 2015, Wuhan had an estimated population of 10,607,700 people. Wuhan is recognized as the political, economic, financial, cultural, educational, and transportation center of central China. It comprises three main boroughs—Wuchang, Hankou, and Hanyang—and these are further divided into seven central and six suburban or rural districts. Jianghan District in Wuhan is chosen as the object of study in this investigation. Located in the middle of Wuhan on the north bank of the Yangtze River, Jianghan District is one of Wuhan’s seven central urban districts and is an important financial, commercial, and trade center for the city. Geographically, this district stretches from latitude 30° 34′ N to 30° 39′ N and from longitude 114°13′ E to 114° 18′ E, and it covers an area of 33.43 sq. km. It accounts for 0.39% of the total area and 15.32% of the developed land of Wuhan City. This region has a registered population of 485,600, and a permanent resident population (including migrant workers) of 710,000 [30]. This region administers 13 sub-districts and 116 community resident committees, as shown in Fig 1. Moreover, this region also administers a few in-city villages (a total of four villages in three sub-districts), including Hejiadun Village and Gusaoshu Village in Hanxing sub-district, Huanzihu Village and Tangjiadun Village in Tangjiadun sub-district, and Hangce Village in Changqing sub-district. The criminal activity in this area is distinctive and many researchers have already applied methods to study the crime patterns in this area, such as the near and near-repeat phenomenon [18] and the local co-location pattern [31].

### 3.2. Study data

The crime data used in this study comprised burglary cases that occurred in Jianghan District, Wuhan City during the eight-month period from January 1 to August 31, 2013. The data are supported by the Public Security Bureau [32] in Wuhan. Each case record contains several attributes, including the type of crime, date of the crime, and location, which is originally recorded as geo-coordinates by the police. In addition, the supporting data for the spatio-temporal hotspot analysis of the crime cases also includes data on the urban administrative divisions (13 sub-districts and 116 communities), data on the urban infrastructure, and the demographic data of Jianghan District in 2013. Each demographic record includes items such as ID number, sex, nationality, date of birth, occupation, and residential address. Table 1 summarizes the data used in the study.

**Table 1:** Description of data attributes.

Dataset	Date Type	Year	Main Attributes
Case data	Point	2013	Type of case, date, and location
Administrative boundaries	Polygon	2010	sub-district and community
Population	Point	2013	Sex, date of birth, occupation, address
POI	Point	2013	Locations of internet bars, hotels, buildings, and residential zones

## Methods

### 4.1 Spatio-temporal Bayesian model

In the spatio-temporal Bayesian model, as the period of time in this study is very short, the temporal effect is considered as linear. This is beneficial in determining developing trends. While involving the spatial effect, the model considers the structured spatial random effect to reveal the interdependence between different regions. Such associated information serves to smoothen and stabilize the estimation results. Hence, this study built a structured spatial random effect by capitalizing on the community-level spatial adjacency relationships. Taking burglary as an example, we assume that the crime rate is *p*_*it*_ in the *i*-th (*i* = 1, 2, …, *N*) community in the *t*-th month (*t* = 1, 2, …, *T*). When the crime rate is not low, the number of crime cases *Y*_*it*_ in the spatio-temporal region obeys a Binomial distribution as follows:

$$Y_{it}∼Binomial(n_{it},p_{it}),$$ (1)

where *n*_*it*_ indicates the number of potential burglary targets in the *i*-th community in the *t*-th month and is represented by the total population of the community. Here, we assume that the total population is not time varying. The logit connectivity function is used to connect crime rate *p*_*it*_ with other relevant factors as follows:

$$logit(p_{it})=\alpha +u_{i}+s_{i}+\gamma {time}_{t}+\delta_{i}{time}_{t}.$$ (2)

In general, the model consists of three components: AREA, TIME, and AREA·TIME. It connects the crime rate with the spatial effect, temporal effect, and spatio-temporal interaction effect. As shown in Eq (2), (*α* + *u*_*i*_ +*s*_*i*_) represents AREA, *γtime*_*t*_ indicates TIME, and *δ*_*i*_*time*_*t*_ denotes AREA·TIME. In detail, α indicates the logarithm of the mean relative crime risk, *u*_*i*_ indicates the spatial unstructured random effect, *s*_*i*_ indicates the spatial structured random effect, *γtime*_*t*_ indicates the temporal effect (time-varying trend of general crime rate), and *δ*_*i*_*time*_*t*_ indicates the spatio-temporal interaction effect in area *i* and time period *t*, reflecting the regional difference in crime rate based on the general development trend.

When the crime rate in the region is relatively low, the number of crime cases *Y*_*it*_ obeys a Poisson distribution as follows:

$$Y_{it}∼Poisson(\lambda_{it}),$$

$$E(Y_{it})=\lambda_{it}=e_{it}\theta_{it},$$ (3)

where *e*_*it*_ indicates the expected number of burglary cases in the *i*-th community in the *t*-th month, and *θ*_*it*_ indicates the ratio of the actual number of burglary cases to the expected number of burglary cases in *i*-th community in the *t*-th month, namely, the relative risk of burglary. Variable *θ*_*it*_ is another variable that is important to analyze.

In the general form of a Bayesian model, the spatial pattern of crimes is usually correlated with socio-economic factors [11]. To control this problem, the fixed effect *βX*_*i*_ may be added to this model. Here, *X*_*i*_ indicates a possible factor correlated with the crime rate (e.g., the unemployment rate, number of hotels, or internet bars), and *β* indicates the regression coefficient of the correlated factor. Taking the binomial distribution as an example, the final form of the model is expressed as follows:

$$logit(p_{it})=\alpha +\beta X_{i}+u_{i}+s_{i}+\gamma {time}_{t}+\delta_{i}{time}_{t},$$ (4)

where *γtime*_*t*_ represents the purely temporal term, which describes temporal variation of crime risk where a mean linear time trend *γ* is assumed; and *δ*_*i*_*time*_*t*_ represents the spatio-temporal interaction term, which expresses the variation of time trends across areas.

### 4.2 Spatial-temporal Bayesian model-based predictive distribution

For a model based on Bayesian theory, the estimation or prediction of the future observed values (e.g., crime rate) depends on the calculation of the predictive distributions [33]. Assume that the parameter set in the model is θ. In the absence of observational data, the estimation of the future observed value *y* is based on the following marginal likelihood function:

f(y)=∫f(y|θ)f(θ)dθ. (5)

Eq (5) averages all possible parameter values, and the results are referred to as the prior predictive distributions. After the observed values of *n* periods (e.g., the data about crime rate *y* over the first *n* months) are obtained, posterior predictive distributions are used to estimate the crime rate of the next period *y*′ as

$$f(y^{'}|y)=\int f(y^{'}|\theta )f(\theta |y)d\theta .$$ (6)

The posterior predictive distributions are the results obtained by averaging all possible values of posterior parameter distributions as *f*(*θ*|*y*). By calculating the posterior predictive distributions, it is possible to infer the future observed values according to the existing data and determine the most likely values and their uncertainty by calculating their mean values and variances.

### 4.3 Parameter estimation and verification

Before this model is used for statistical inference, it is necessary to set prior distributions for unknown parameters. Specifically, both the correlation coefficient *β* and temporal trend *γ* obey a normal distribution with a mean of 0 and variance of 1,000. The logarithm of the mean relative crime risk *α* obeys a uniform distribution over the range [0, 100]. The distributions of spatial structured random effect *S*_*i*_ and spatio-temporal interaction effect *δ*_*i*_ are given by intrinsic conditional autoregressive Gaussian distributions [8]. Under this condition, the mean values of *s*_*i*_ and *δ*_*i*_ depend on the corresponding values of the adjacent regions through the adjacency matrix. Unstructured spatial random effect *u*_*i*_ obeys an independent identical normal distribution with a mean value of 0. The standard deviation of the prior distributions of all spatial random effects are set through three unknown parameters (*σ*_*s*_, *σ*_*δ*_, and *σ*_*u*_) with a uniform prior distribution over the range [0, 100].

The model was fitted using a software named WinBUGs, which implements the model and Markov chain Monte Carlo (MCMC) algorithm to generate dependent samples based on the posterior distribution of the model. Methods of sample generation for generating the target posterior distribution of each parameter by MCMC in WinBUGS have been reported extensively in the literature [20,21]. The model fit and identification of the final (better) model was evaluated using the deviance information criterion (DIC) [34]. The DIC is a generalization of the Akaike information criterion for evaluating Bayesian hierarchical modeling and has been the most widely used statistic for comparing the fitness of Bayesian spatial models. It assesses the model fit by analyzing deviance and model complexity. It is defined as

$$DIC=\bar{D}+\rho_{D},$$ (7)

where $\bar{D}$ is the posterior mean of the deviance, and *ρ*_*D*_ is the number of effective parameters in the model. The model with smaller DIC value is considered to be a better choice. Thus, for the Poisson and Binomial distribution used in the spatial-temporal Bayesian model, this research will compare the DIC values to select an appropriate distribution for predicting crime events.

## Results

### 5.1 Exploratory data analysis

The crime cases selected for analysis in this study are burglaries, which fall under the category of frequent offences against property. Burglaries are closely correlated with population distribution, and their detection rate is very low. Burglaries are a type of crime to which the law enforcement authorities pay much attention. They occur with higher frequency than other types of crime events, and tend to converge spatio-temporally; specifically, they are very likely to have spatio-temporal hotspots. The analysis of the spatio-temporal characteristics and patterns of burglary cases will provide scientific evidence for the effective prevention and control of these crimes. It further enables police patrols to be highly targeted, thus enabling law enforcement to tackle crime effectively.

In the period covered by the study, a total of 1,346 burglary cases occurred. This study first analyzes the change in the number of burglary cases over time, determines the total number per month, and calculates the average daily number of burglary cases for each month to eliminate any influence of month length and facilitate comparisons among months. Table 2 shows the number of burglary cases over time. It can be seen that the number of burglary cases is the lowest in February (4.5 cases per day on average). This may be because China’s traditional Spring Festival usually occurs in late January or early February. During this period, there is a substantial population movement, particularly the return of non-local workers to their hometowns. This is consistent with the conclusions obtained in previous studies about urban burglaries [35]. Overall, the number of burglary cases is relatively steady. In community-level statistics, the number of communities whose case number is larger than zero in each month ranges between 60 and 70, indicating that almost 60% of communities experience burglaries. Furthermore, the maximum number of burglary cases in each community is calculated; February is still the month having the lowest value. Examining the records, we find that the Huaanli community has the largest burglary cases each month, except in February. Therefore, it should be one of the most important surveillance areas for the police.

**Table 2:** The statistics results of burglary cases in communities.

Month	Burglary	Burglary per Day	Community (Case No. >0)	Burglary per Community	Min	Max
**1**	**182**	**5.87**	**69 (**59.48%**)**	**1.57**	0	20
**2**	**127**	**4.56**	**55 (**47.41%**)**	**1.09**	0	7
**3**	**179**	**5.77**	**66 (**56.89%**)**	**1.54**	0	21
**4**	**174**	**5.8**	**68 (**58.62%**)**	**1.5**	0	12
**5**	**156**	**5.03**	**61 (**52.58%**)**	**1.34**	0	18
**6**	**162**	**5.4**	**64 (**55.17%**)**	**1.39**	0	11
**7**	**207**	**6.68**	**72 (**62.17%**)**	**1.78**	0	19
**8**	**159**	**5.12**	**68 (**58.62%**)**	**1.37**	0	22

**Fig 2:**
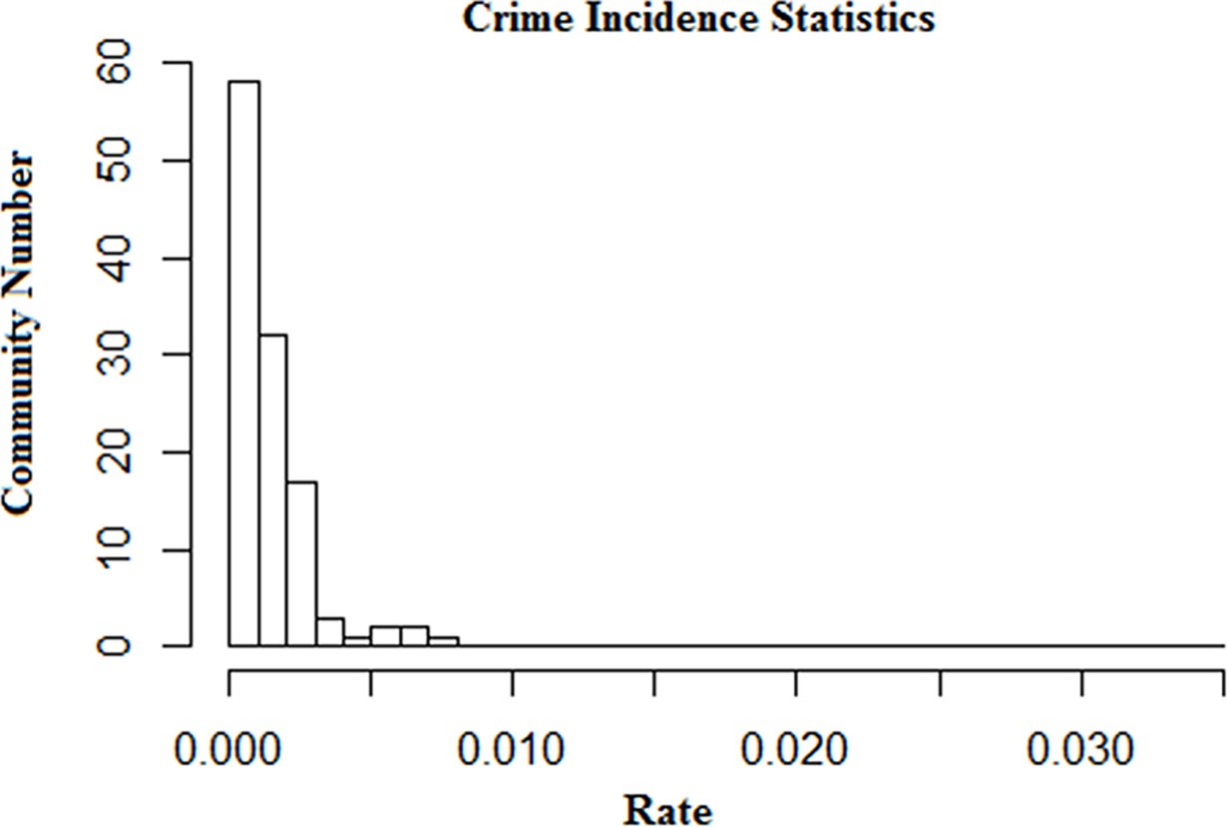
Crime incidences of communities.

The distribution of the case locations only reflects the distribution of their density. To conduct a descriptive analysis of crime risk from the perspective of communities, it is necessary to consider the resident population of each community. This study calculated the resident population of each community according to the population data and defined the community crime rate as the number of crime cases per community population. Fig 2 shows the incidences of burglaries in the communities. Crime incidence refers to the number of crimes that have occurred in a given area, and it is usually expressed as a rate per head of population [36]. Here, the unit is one crime case per 1000 population. Overall, the community crime incidence decreases exponentially. The number of burglary cases per 10,000 population is smaller than 27 in 90% of the communities, while it is larger than 50 in only five communities.

**Fig 3:**
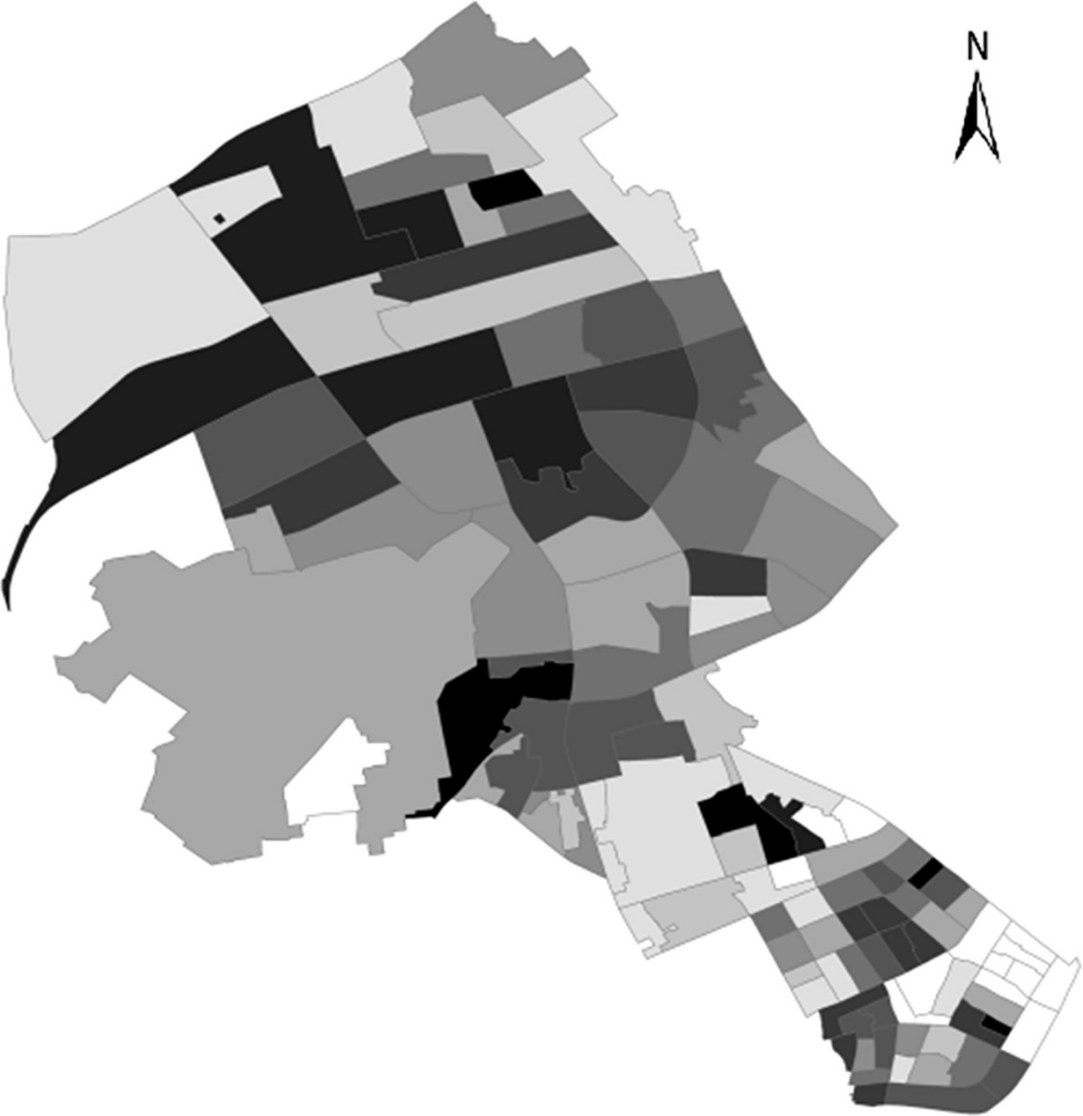
Distribution of burglary incidence in districts.

Fig 3 shows the spatial distribution of the community crime incidence. The areas with high burglary crime incidences are mainly distributed in the middle and northern areas. The top 10 communities with the highest burglary crime rate are Wangjiadun Community, Shengyun Community, Changjian Community, Shicai Community, Chang’er Community, Jingwu Community, Yulanli Community, Huaanli Community, Yangguang Community, and Yinheli Community. The targets of burglary crimes show that the distribution of burglary cases may have close relations with the distribution of residential communities.

### 5.2 Hotspot analysis of crime trends

Crime rate is closely related to unemployment. The decline in property crime rates is attributable to the decline in the unemployment rate. Different crimes in different areas varied in terms of the severity of crimes. The police department in our research area revealed that, for burglary, criminals prefer to detect the environment of communities to figure out the best target, including the risk, and wealth level of a residential zone. Different from countries such as the U.S., the residential zone in urban cities of China are enclosed by gates and walls or fences, which are protected by guards. Some areas are easy to enter and exit, while some are not. In a community, there are many isolated residential zones. Hence, it is important for criminals to fully know their targets. Furthermore, before the crime, criminals prefer to stay at hotels or internet bars, which have less supervision and are close to target areas, to study the environmental dynamics. Therefore, in this study, five key factors relevant to burglary are considered in the spatial-temporal Bayesian model as shown in Eq (5), including hotel (*X*_1_), internet bar (*X*_2_), business building (*X*_3_), residential zone (*X*_4_), and unemployment (*X*_5_) in each community; thus, *X* = (*X*_1_,*X*_2_,*X*_3_,*X*_4_,*X*_5_). In order to keep the consistency of variables, *X*_1_, *X*_2_, *X*_3_, *X*_4_, and *X*_5_ are converted to the number per population in each community. Meanwhile, in Eq (5), *β* = (*β*_1_,*β*_2_,*β*_3_,*β*_4_,*β*_5_) indicates the linear coefficient value of each variable.

To analyze hotspots and determine crime trends, we first evaluated the spatio-temporal Bayesian model with a binomial distribution using Eqs (1) and (5). We then used the MCMC approach to sample the posterior distributions of the parameters. We set the number of annealing iterations to 50,000 after conducting 100,000 iterations, and use the residual samples for statistical inference. To reduce the dependency among samples, the dilution ratio was set to 50:1; in particular, we drew one out of 50 samples for use as a final sample. Finally, we obtained the main parameter estimation results of the model according to the posterior distribution samples, as listed in Table 3.

**Table 3:** Main parameter estimation results of the binomial distribution model.

Parameter	Mean	Standard Error	MC Error	p = 2.5%	Median	p = 97.5%	Z-test
α	−10.070	0.248	0.0133	−10.540	−10.060	−9.591	−0.07
beta1	0.009	0.014	0.0004	−0.018	0.009	0.036	−1.31
beta2	0.163	0.052	0.0021	0.061	0.165	0.264	−0.42
beta3	0.013	0.032	0.0009	−0.050	0.014	0.077	0.23
beta4	0.134	0.036	0.0013	0.060	0.133	0.205	1.20
beta5	3.930	2.489	0.1302	−0.845	3.864	8.872	−0.30
γ	0.010	0.015	0.0005	−0.019	0.011	0.040	−1.45
δ (s.d.)	0.128	0.035	0.0015	0.059	0.127	0.199	−0.30
*s* (s.d.)	1.203	0.268	0.0137	0.670	1.207	1.710	−1.17
*u* (s.d.)	0.367	0.159	0.0095	0.065	0.369	0.676	1.76

Note: s.d. = standard deviation.

We also investigated the Poisson distribution settings for the model (Eqs (4) and (5)). Similarly, we used the MCMC approach to sample the posterior distributions of the parameters, where the annealing iterations were set to 150,000 after conducting 200,000 iterations. We used the residual samples for statistical inference and set the dilution ratio to 50:1. Tables 3 and 4 present the final main parameter estimation results of this model.

**Table 4:** Main parameter estimation results of the Poisson distribution model.

Parameter	Mean	Standard Error	MC Error		p = 2.5%	Median	p = 97.5%	Z-test
α	−1.341	0.269	0.0360	−1.849		−1.349	−0.800	0.504
beta1	0.008	0.014	0.0014	−0.019		0.007	0.037	1.685
beta2	0.164	0.056	0.0069	0.057		0.165	0.276	−0.553
beta3	0.007	0.034	0.0034	−0.062		0.008	0.073	0.653
beta4	0.143	0.031	0.0029	0.085		0.142	0.206	−1.659
beta5	4.160	2.513	0.3223	−0.553		4.211	9.254	−0.172
γ	0.011	0.017	0.0012	−0.021		0.011	0.043	−1.105
δ (s.d.)	0.127	0.038	0.0044	0.056		0.125	0.201	0.374
*s* (s.d.)	1.297	0.173	0.0230	0.966		1.294	1.664	−0.447
*u* (s.d.)	0.331	0.128	0.0173	0.098		0.329	0.582	−0.914

Note: s.d. = standard deviation.

The estimation results of the Poisson distribution-based model are basically the same as those of the binomial distribution-based model. Table 5 compares the DIC values of the binomial distribution model and Poisson distribution model. The model with a smaller DIC value is considered to be better. *D* is the posterior mean of the deviance, ***D***(***θ***) is the deviance of the posterior means obtained by using the posterior means of the relevant parameters to calculate the deviance, and ***p***_***D***_ is the number of effective parameters in the model. The DIC value of the binomial distribution model is slightly smaller, and thus the two models have the same effectiveness. This study uses the estimation results of the binomial distribution model for further analysis.

**Table 5:** DIC evaluation of the models.

Model	*D*	*D*(*θ*)	*p*_*D*_	DIC
Binomial distribution model	2,360.03	2,253.92	106.106	2,466.13
Poisson distribution model	2,357.98	2,249.65	108.336	2,466.32

**Fig 4:**
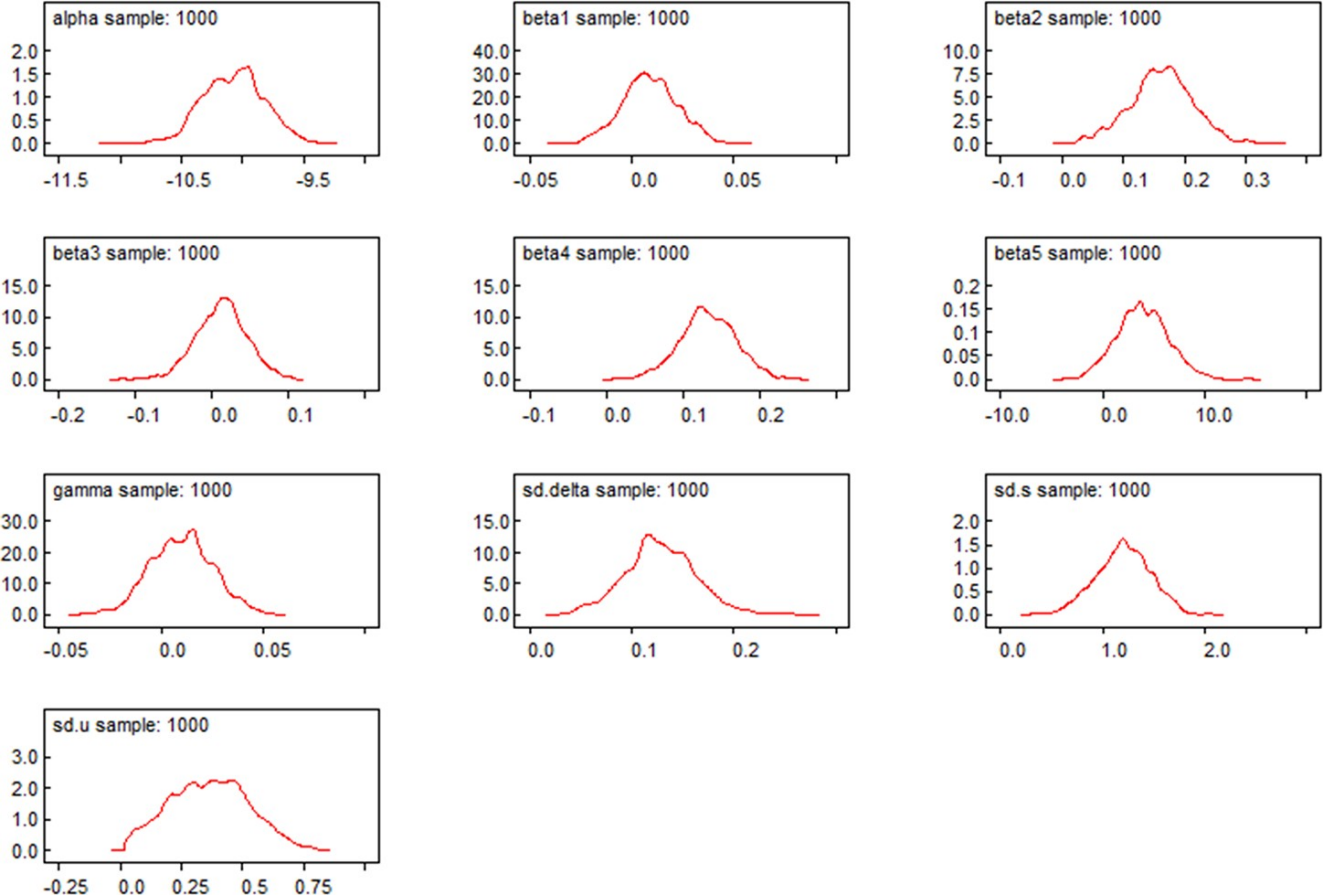
Kernel density estimation results of the sample distribution of the parameters.

**Fig 5:**
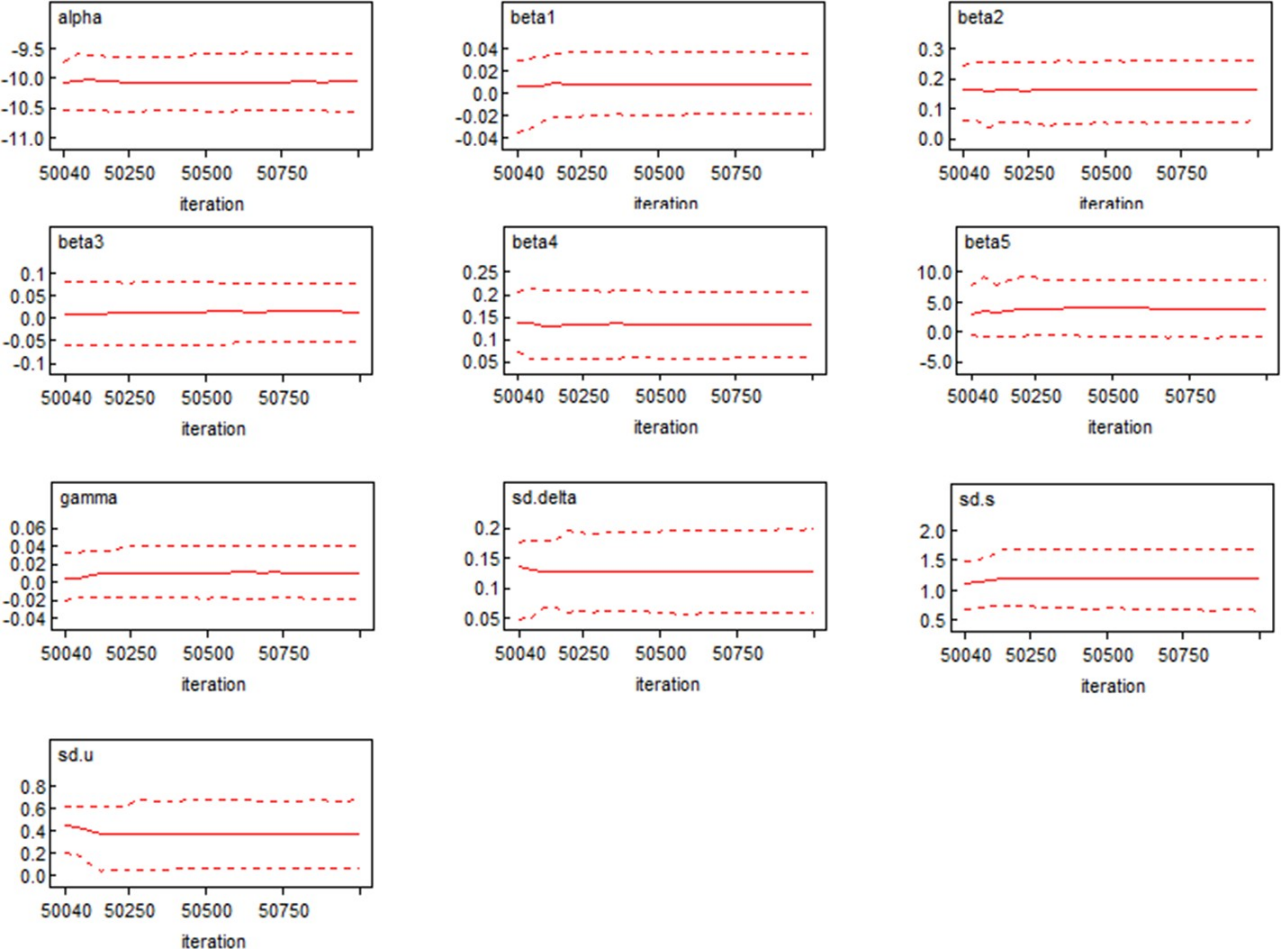
Quantile sequence diagrams of the parameter iteration sequences.

**Fig 6:**
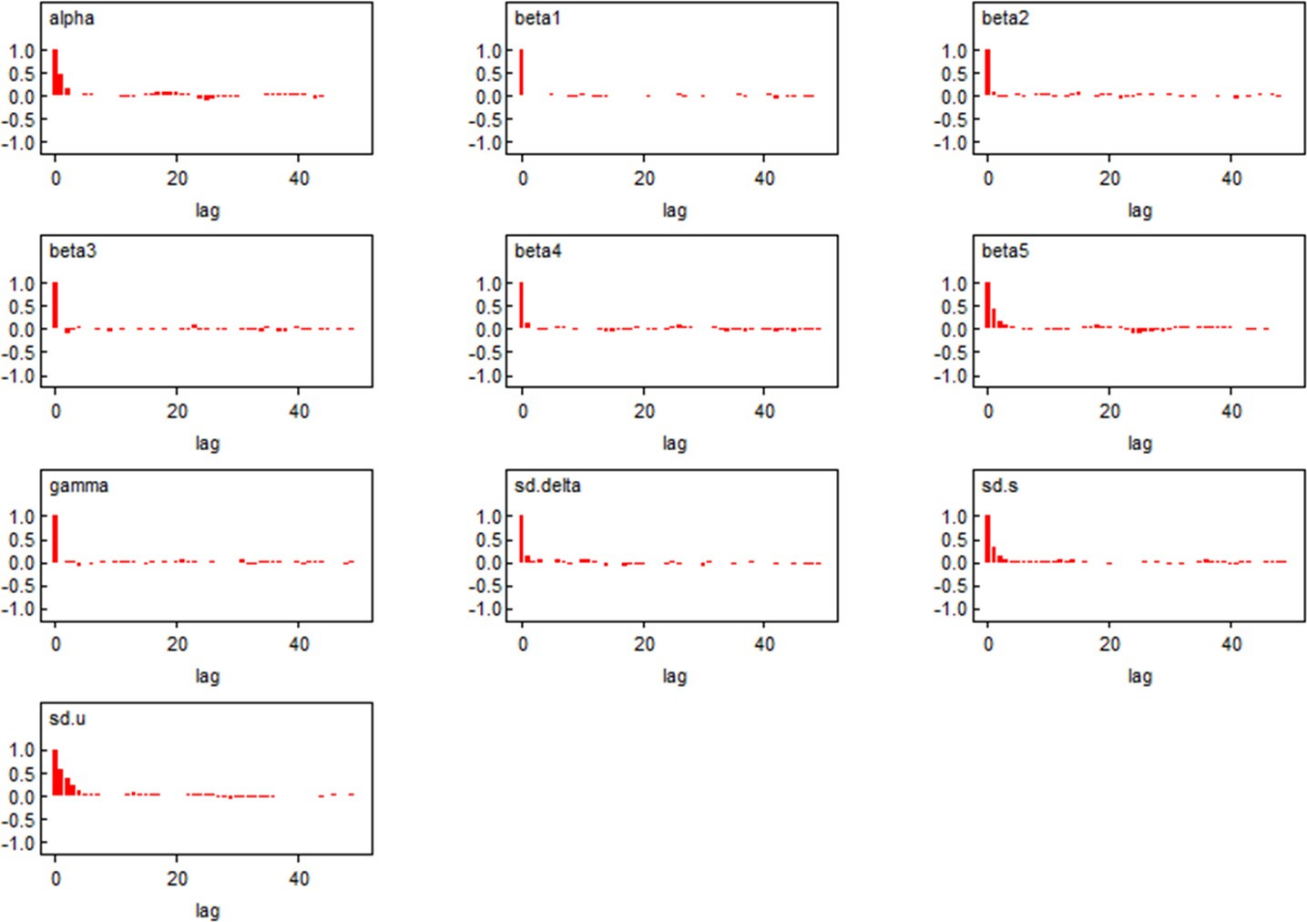
Autocorrelation sequences of the parameters.

Fig 4 shows the kernel density estimation [37] results of the sample distribution of the parameters. All parameters approximately obey a symmetric distribution; their mean value and median value are all similar (the same as the Mean and Median column shown in Tables 3 and 4) and their Monte Carlo errors are far smaller than their standard errors. This shows that the estimation results of the posterior distributions are very reliable, and their mean values can be used as the final estimated values of the parameters. Fig 5 presents the 2.5%, 50%, and 97.5% quantile sequence diagrams of the parameter iteration sequences. The three quantile sequences all tend to be smooth and steady, and it preliminarily appears that the iteration sequences are convergent. Fig 6 shows the autocorrelation sequence chart. For the samples with a first-order lag, the significance is reduced significantly. For the samples with a third-order lag, the autocorrelation coefficient is approximately zero, indicating a high independence among the samples.

According to the parameter estimation results in Table 3, the mean value of coefficient beta2 (the number of internet bars) and beta4 (the number of residential zones) are 0.163 and 0.134, respectively, while the credible interval values are (0.061, 0.264) and (0.06, 0.205), respectively, indicating that these two variables are statistically significant at the confidence level of 0.05. The estimated coefficient values of other parameters (including the number of hotels, number of business buildings, and unemployment rate) are all not statistically significant. Specifically, the estimated coefficient value of the unemployment rate is 3.93, which is significant at the confidence level of 0.1. Table 4 shows the main parameter estimation results. These results demonstrate that the community burglary rate is primarily positively correlated with the number of internet bars and residential zones in each community, and is secondarily positively correlated with the unemployment rate but is not correlated with the number of hotels and number of shopping and office buildings in each community. This leads to the conclusion that burglaries are very likely to occur in residential zones and the relationship between crime rate and the average number of population per internet bar may exist because crime suspects often use internet bars as temporary hideouts or they prefer to stay there as part of their routine activities to obtain better information about nearby residential zones. Owing to its loose management, internet bars have been proved to be one of the most popular locations for criminal offenders to execute crime-related activities [38].

The estimated mean for temporal effect parameter γ is close to 0.010. Its credible interval is (−0.019, 0.04) and the estimation result is not statistically significant at the confidence level of 0.05. This shows that the time-varying community crime rate is overall steady during the eight months.

The crime rate with respect to time is jointly determined by intercept term (*α* + *u*_*i*_ + *s*_*i*_) and slope (*γ* + *δ*_*i*_). Structured spatial random effect *s*_*i*_, unstructured random effect *u*_*i*_, and spatio-temporal random effect *δ*_*i*_ are all parameters associated with spatial random effects. The estimation results of their variances are all significant at the confidence level of 0.05: *s*_*i*_ (1.203, credible interval (0.67, 1.71)), *u*_*i*_ (0.367, credible interval (0.065, 0.676)), and *δ*_*i*_ (0.128, credible interval (0.059, 0.199)). The variance of *s*_*i*_ is far larger than the variances of *u*_*i*_ and *δ*_*i*_, indicating that spatial correlation plays a dominant role in influencing the difference in crime rates in the community. The significance level of *δ*_*i*_ shows that the developing trends of crime rate vary significantly from community to community.

**Fig 7:**
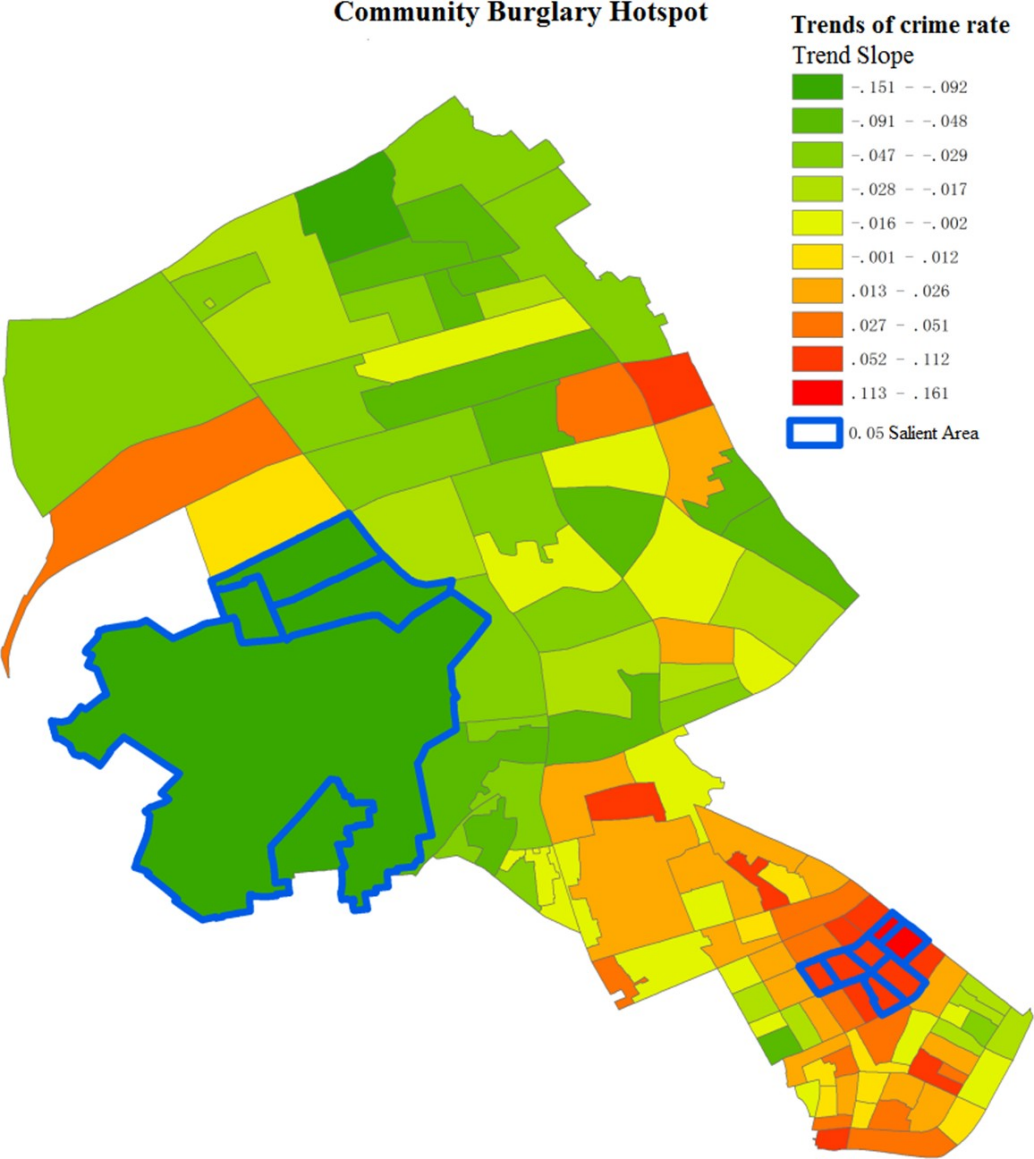
Burglary rate hotspots.

**Fig 8:**
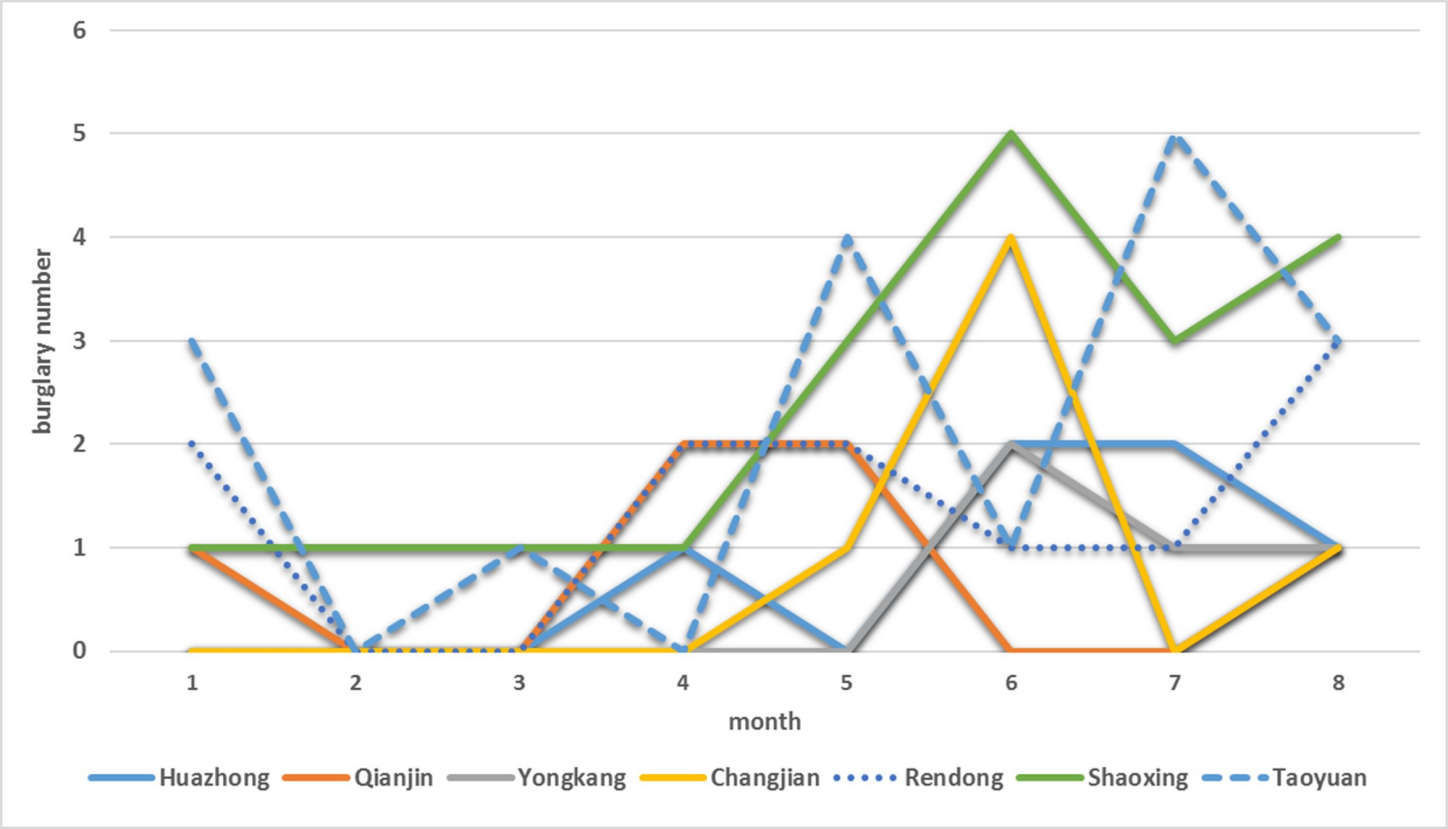
Numbers of burglaries over time in the hotspots.

Fig 7 shows the distribution of development trend *δ*_*i*_ for burglary rate in each community. Overall, the areas to the north of Jianshe Avenue of Jianghan District are coldspots. Specifically, the salient coldspots include Changhong Community, Fuxing Community, and Yangzi Community in Changqing Sub-district, and Jichang Community in Wansong Sub-district. In these areas, the number of burglary cases is very low, and is overall decreasing. In the northern areas, Huaanli Community in Hanxing Sub-district is an unexpected hotspot. Most hotspots are distributed in Shaoxing Community, Rendong Community, and Taoyuan Community in southern Qianjin Sub-district, as well as Qianjin Community, Yongkang Community, Changjian Community, and Huazhong Community in Hualou and Shuita Sub-districts. This can also be verified by the number of actual burglary cases over time (shown in Fig 8). Despite many fluctuations, each salient hotspot shows an increase in the burglary rate. Comparing spatial hotspots and near-repeat hotspots, there exist hotspots in three communities in Qianjin Sub-district and Huaanli Community in Hanxing Sub-district. Special prevention and control measures should be taken in these areas, such as increasing police patrol times, especially at night.

**Fig 9:**
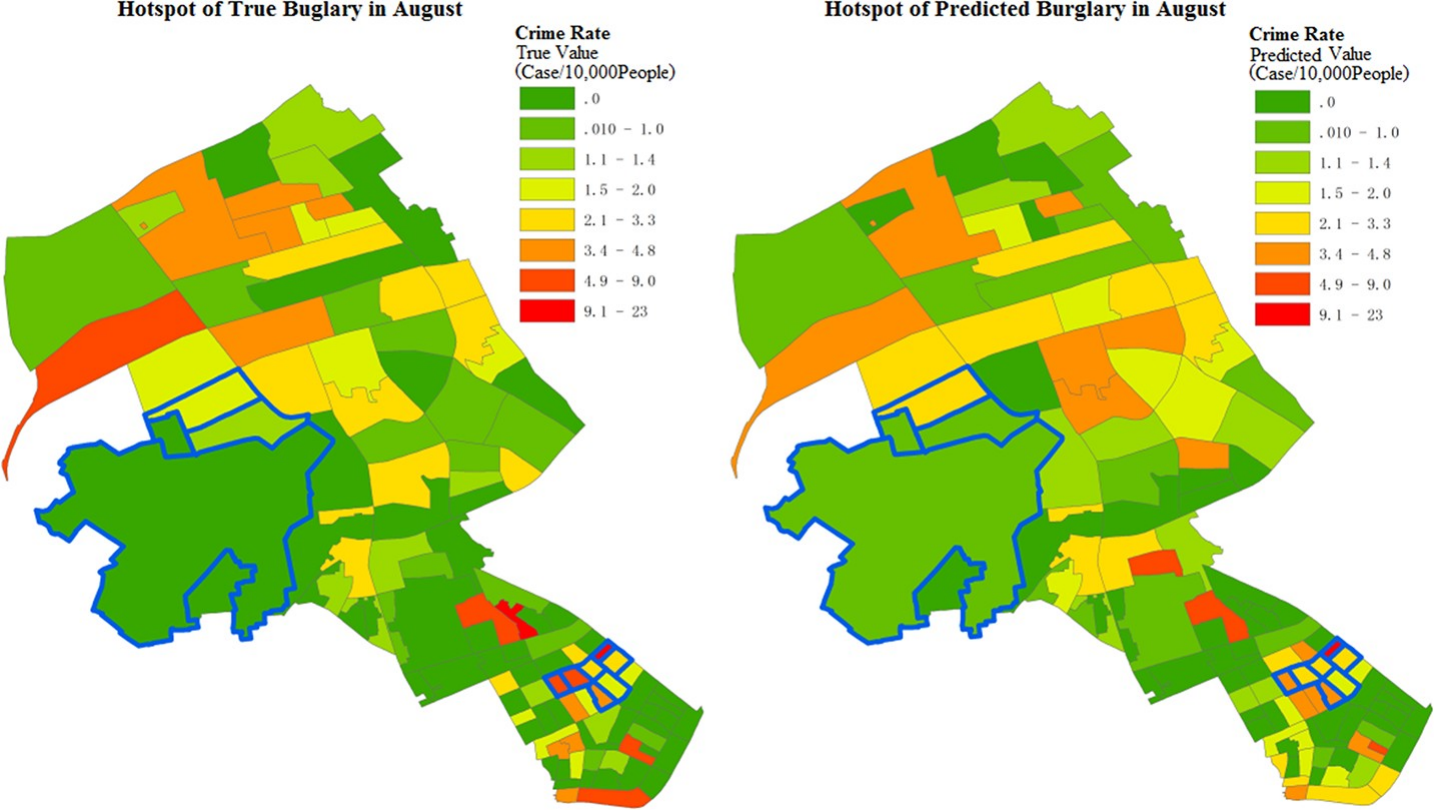
Distribution of true and predicted burglary crime rates in August. (a) True crime incidence and (b) Predicted crime incidence.

Further, this study used the community crime data of the seven previous months as test data. Using the prediction distributions, the number of crime cases and crime rate of 116 communities in August were statistically inferred and the predicted results were compared with the actual results. Table 6 lists the estimated results for the hotspots. For communities with a significant increase in crime rate, the large standard deviations indicate a substantial fluctuation in the predicted values, but the deviation between the predicted and actual medians is very small. Fig 9 shows the spatial distribution of the actual and predicted values for community crime rate in August. These results show that the predictive distributions can reflect the overall distribution function of the actual crime rate.

**Table 6:** Estimated results of significant hotspots.

Community	Predicted Average Number	Predicted Median	Actual Case Number	Standard Deviation	p = 2.5%	p = 97.5%	
Huazhong	1.6	1	1	1.538	0	5	
Changjian	1.3	1	1	1.391	0	5	
Shaoxing	3.253	3	4	2.176	0	9	
Qianjin	1.368	1	1	1.307	0	4	
Yongkang	0.785	1	1	0.9656	0	3	
Rendong	1.632	1	3	1.449	0	5	
Taoyuan	3.023	3	3	2.099	0	8	

## Discussion and conclusion

Spatio-temporal Bayesian modeling, which is based on regional statistics, is widely used in epidemiological studies. This approach represents an improvement on the traditional group-based trajectory analysis method. Considering the spatio-temporal random effect and relevant factors, the Bayesian model enables a combined analysis of spatio-temporal correlation and crime causes to be conducted. Since spatio-temporal correlation is taken into account, this approach can consider the influence of associated areas when predicting the crime rate. This approach is suitable for estimations over local regions. However, the statistical inference of the Bayesian model is based on the samples of the posterior distributions; hence, the statistical inference involves calculations for convergence and is also influenced by the sampling quality. To ensure the robustness of the Bayesian model, the number of sampling times should be as high as possible. This calls for a highly efficient model, and this should be considered in practice.

To address the increasing crime rate hotspots, this study built a spatio-temporal Bayesian model specific to the community crime rate. This spatio-temporal Bayesian model considered the spatial correlation and possible influencing factors of crime rate and was used to analyze the spatio-temporal distribution and developing trends regarding crime rate. The analysis results show that of the possible relevant factors, the number of internet bars and hotels in each community, are positively correlated with the community burglary rate. The positive correlation between the average number of internet bars may be because crime suspects use internet bars to survey crime sites beforehand. There are several features regarding internet bars in our research area: (1) Loose supervision of internet bars allows any kind of customer to enter and exit without much restriction. (2) Besides nearby students, many unemployed or low-income customers prefer to stay the whole day in internet bars because of cheaper price compared to hotels. Thus, the internet bar is one of the best places to hide potential criminals looking for financial gain. It is important for the local public security bureau to enhance the supervision level for internet bars, especially “black internet bars” without legal business license. Moreover, identification, registration, and verification of any customer staying at the internet bar should be made mandatory so as to enable police to track the trajectory of potential criminals.

The estimation results for the developing trends of community crime rate show that the coldspots for burglary rate are mainly distributed in the areas to the north of Jianshe Avenue in the middle of Jianghan District, while the hotspots are distributed in Qianjin, Hualou, and Shuita Sub-districts in the southern areas of Jianghan District. The estimation results for hotspots can be used as a reference for determining the key areas for crime prevention and control in the short term. Considering the impact factors of burglaries, the internet bars in these hotspot sub-districts are the most important supervision targets. Thus, this could be used to plan how police units should be deployed, such as increasing patrolling and random checking times for prominent internet bars.

However, there are limitations to this study. First, the original data provided by Wuhan Public Security Bureau only recorded the exact time of burglary without considering the condition that burglary happens when people are not at home. Thus, the recorded time is not accurate enough, and it is better to use other complementary information to clean the temporal data, such as average time method, rigid temporal search method or aoristic search method [39]. Second, to normalize the variables in the model, the community population is considered as the denominator, while other studies use households as the denominator [40]. This is because we are unable to obtain the households data. In the future, when the data is collected, both denominators should be analyzed and compared. Third, for the Bayesian model in this study, the time period is short, so the temporal effect is considered as linear; this is beneficial for determining the developing trends and impact factors of crime. For long-term fine-granularity data, the Bayesian model should consider the periodical and non-linear trends over time; one solution is to build a multi-order time sequence autoregressive model. Other non-linear models can also be employed to predict crimes, such as neural network model and deep learning model. However, since more samples are needed, it is difficult to explore and explain the relationship between variables and results.

In this study, there are many zero crime communities, as shown in Table 2. Thus, it is possible to adopt zero-inflated models in future work, such as zero-inflated Poisson (ZIP) model [41] and zero-inflated binomial (ZIB) model [42], which generally have two zero generating processes. The first process is governed by a binary distribution that generates structural zeros. The second process is governed by a Poisson or negative binomial distribution that generates counts. The models have been well applied in public health [43,44], when the zero frequency is over 80%. Additionally, the hierarchical structure (e.g., Level 1 for sub-district data, and Level 2 for community data) of the spatial data and real adjacency relationship between communities regarding crime rate should be explored. When analyzing the relevant factors, it is necessary to collect the related data and thus analyze additional potential factors associated with the crime rate considering the environmental criminology, so that a priori knowledge can play a substantial role. The final goal of future work is to construct a Bayesian model that estimates the crime rate more accurately by better utilizing the spatio-temporal crime rate distribution and relevant factors.
